# Translational biomarkers of acetaminophen-induced acute liver injury

**DOI:** 10.1007/s00204-015-1519-4

**Published:** 2015-05-17

**Authors:** Richard D. Beger, Sudeepa Bhattacharyya, Xi Yang, Pritmohinder S. Gill, Laura K. Schnackenberg, Jinchun Sun, Laura P. James

**Affiliations:** Division of Systems Biology, National Center for Toxicological Research, Food and Drug Administration, 3900 NCTR Road, Jefferson, AR USA; Department of Pediatrics, University of Arkansas for Medical Sciences, Little Rock, AR USA; Clinical Pharmacology and Toxicology Section, Arkansas Children’s Hospital, Little Rock, AR USA

**Keywords:** Acetaminophen, MicroRNA, Proteomics, Metabolomics, Biomarkers

## Abstract

Acetaminophen (APAP) is a commonly used analgesic drug that can cause liver injury, liver necrosis and liver failure. APAP-induced liver injury is associated with glutathione depletion, the formation of APAP protein adducts, the generation of reactive oxygen and nitrogen species and mitochondrial injury. The systems biology omics technologies (transcriptomics, proteomics and metabolomics) have been used to discover potential translational biomarkers of liver injury. The following review provides a summary of the systems biology discovery process, analytical validation of biomarkers and translation of omics biomarkers from the nonclinical to clinical setting in APAP-induced liver injury.

## Introduction

Acetaminophen (APAP), also known as paracetamol and *N*-acetyl-p-aminophenol, is a drug commonly used for pain relief and fever reduction. Acetaminophen is generally safe at the recommended doses, although large doses of APAP that are greater than the recommended dose can cause liver necrosis (McJunkin et al. 1976), liver failure and death (Larson et al. 2005; Lee 2008). A number of mechanisms have been linked to the development of liver injury, including glutathione depletion (James et al. 2003; Mitchell et al. 1973; Vendemiale et al. 1996), oxidative stress (Jaeschke et al. 2003), formation of reactive oxygen species (ROS) (Hinson et al. 2004; Michael et al. 1999), formation of reactive nitrogen species (RNS) (Hinson et al. 2004), mitochondrial dysfunction (Kon et al. 2004) and disruption of energy metabolism (Chen et al. 2009; Coen et al. 2003). In the clinical setting, acetaminophen liver injury covers a spectrum of disease severity, ranging from asymptomatic transient hepatitis to acute liver injury failure, accompanied by coma and death. Acetaminophen is responsible for approximately half of the reported cases of acute liver failure (ALF) in the USA (Larson et al. 2005); between 1998 and 2013, over half of the APAP-induced ALF cases were unintentional (Lee 2008). The current diagnosis of APAP overdose is based on elevation of APAP levels in peripheral blood (Rumack et al. 1981), elevation of the clinical chemistry biomarker, alanine aminotransferase (ALT) and estimation of APAP ingested (Zyoud et al. 2012). ALT is the most commonly used biochemical indicator of liver injury, but lacks specificity (Amacher 1998; Ozer et al. 2008). Patient histories reporting ingestion of toxic does of APAP are known to be unreliable (Dougherty and Klein-Schwartz 2012; Polson et al. 2008), and many patients are unaware of the inclusion of APAP in over-the-counter medications (Wolf et al. 2012) and prescription pain medications. Elevation of APAP levels in peripheral blood within the first 24 h of the APAP overdose is used by practicing physicians to assess the risk of liver injury and the potential need for treatment with the antidote, *N*-acetylcysteine (NAC). A plot of the APAP level in relationship to the stated time of APAP overdose is commonly known as the Rumack nomogram (Rumack et al. 1981). While this approach is widely used in hospital emergency departments and acute-care settings, it has numerous limitations, which include its reliance on the patient’s recognition of APAP overdose and an accurate history of the time of the overdose. In addition, the Rumack nomogram was initially designed and intended for use within the first 24 h of the APAP overdose and is based on anticipated drug clearance following a single, acute ingestion of a toxic dose of APAP (Rumack 2002). A significant proportion of APAP overdoses are known to be chronic in nature and/or associated with long-term exposure to APAP at doses above those recommended by the manufacturer (Daly et al. 2004; Schiødt et al. 1997). Thus, the limitations of current diagnostic approaches and the wide-spread use of this analgesic make it imperative to discover and validate sensitive and specific translational biomarkers of APAP-induced liver injury. The development of systems approaches together with pioneering “omic” technologies and computational tools will lead the way for future translational systems medicine.

### Metabolism of acetaminophen

**Fig. 1:**
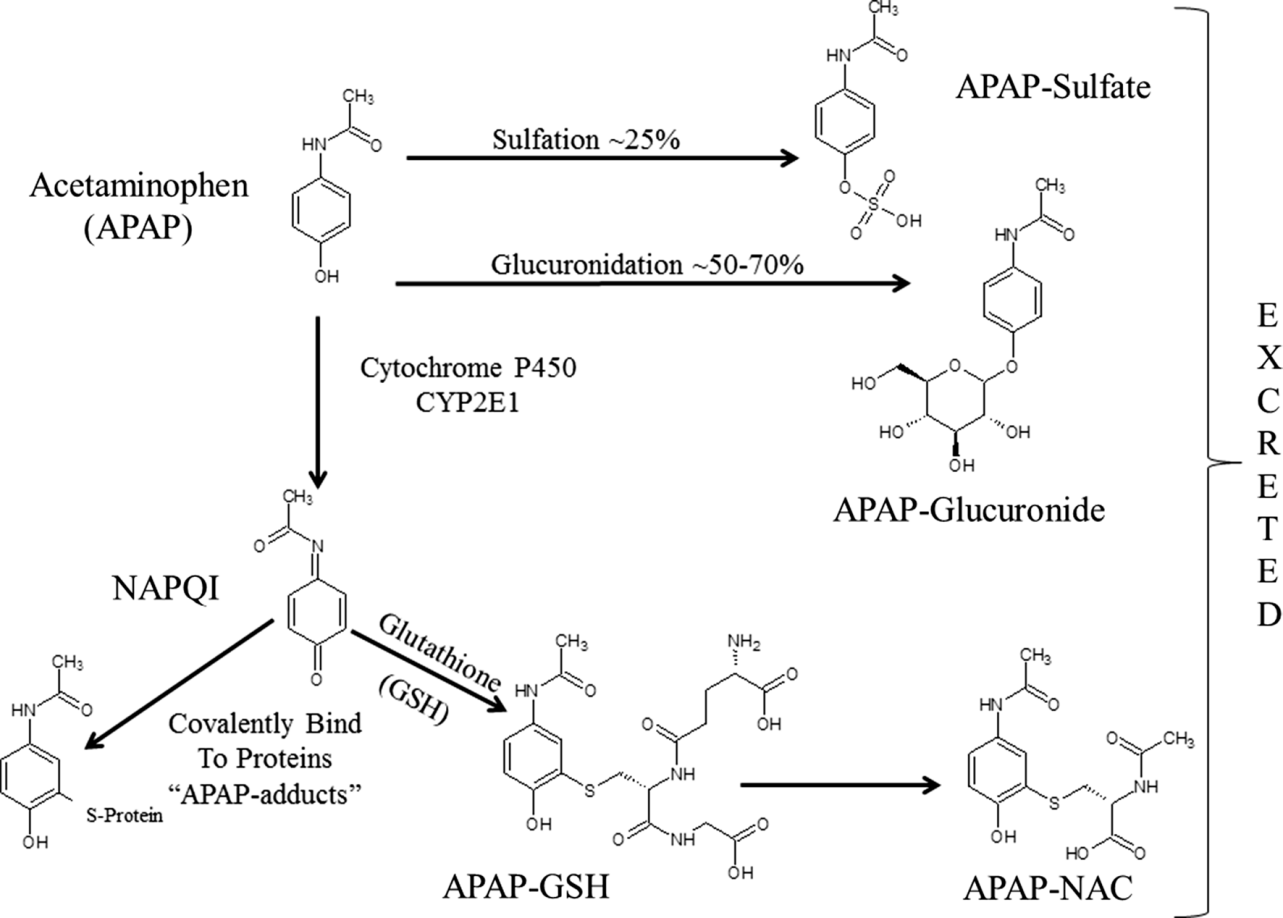
Cartoon depicting acetaminophen phase II metabolism to APAP-sulfate and APAP-glucuronide. APAP is metabolized by CYP2E1 to NAPQI which can be subsequently metabolized to APAP–GSH with the addition of GSH and then metabolized to APAP–NAC

Figure 1 shows the metabolism of APAP. Under normal conditions, APAP is primarily metabolized to the sulfated or glucuronidated forms and then excreted by the kidneys (Bales et al. 1984; McGill and Jaeschke 2013; Watari et al. 1983). A minor portion of the drug is metabolized in the liver by the cytochrome P450 enzymes (primarily CYP2E1) to the reactive and toxic APAP intermediate, *N*-acetyl-p-benzoquinone imine (NAPQI) (Patten et al. 1993). NAPQI can be readily detoxified by conjugation to glutathione and excreted in the glutathione and *N*-acetyl-cysteine forms (Potter and Hinson 1986; Shayiq et al. 1999). The portion of NAPQI that is not detoxified by glutathione can bind to proteins and DNA (Bartolone et al. 1988, 1989). NAPQI bound to cysteine residues in proteins, hereafter referred to as APAP protein adducts, has been observed in tissue and in biofluids (Roberts et al. 1991). APAP adducts have been shown to correlate with ALT levels in APAP overdose in both preclinical and clinical studies. Previous reviews have addressed early work characterizing the relationship of APAP protein adducts to liver injury in nonclinical models of APAP liver injury (McGill and Jaeschke 2013; Roberts et al. 1991). APAP protein adducts have been shown to correlate with ALT levels in APAP overdose in both preclinical and clinical studies (James et al. 2001; Muldrew et al. 2002).

### Definition of biomarker

The FDA defines biomarker as a “characteristic that is objectively measured and evaluated as an indicator of normal biological processes, pathogenic processes or biological responses to a therapeutic intervention” (US  2006, 2010). The FDA defines a prognostic biomarker as a “measured characteristic that reflects a patient’s degree of risk of disease occurrence or progression” that is independent of treatment while a predictive biomarker categorizes patients by their likelihood to respond favorably or adversely to a particular treatment. A diagnostic biomarker is something that can be measured, which is an indication of certain disease state (Beger and Colatsky 2011). Pharmacodynamic (PD) biomarkers are defined as the biological response to a drug treatment (Sawyers 2008) and either are treatment specific or may represent endogenous phenotype changes in a subject due to a drug treatment (Beger and Colatsky 2011). PD biomarkers can be either diagnostic biomarkers or prognostic biomarkers. Translational biomarkers can be defined as biomarkers found in nonclinical studies using the technologies that can be used in the clinic. In this review, we are focusing on translational biomarkers that can be detected in biofluids since biofluids can be easily obtained in the clinic.

### Discovery and validation of biomarkers using omics technologies

**Fig. 2:**
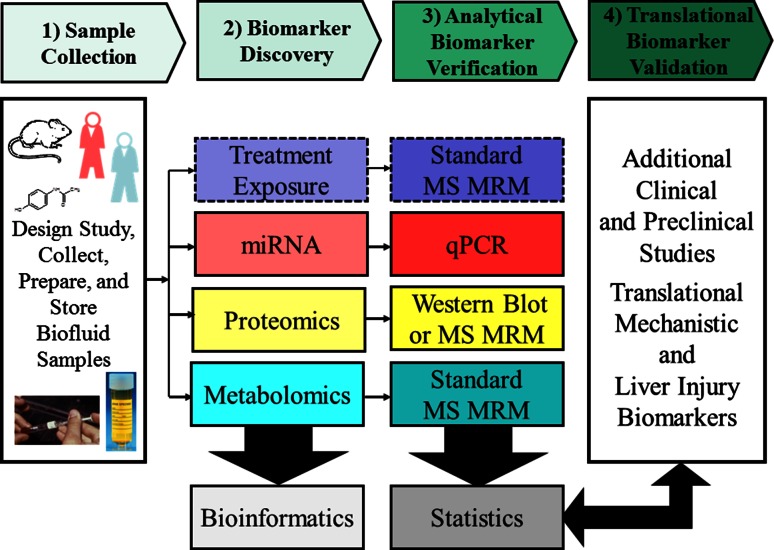
Flow chart showing the process of discovering treatment-specific PD treatment and omics biomarkers, followed by analytical verification and testing of the biomarkers in additional clinical and preclinical studies for translational validation

The goal for new predictive translational safety biomarkers is to be able to monitor early indications of organ toxicity in clinical trials, so better informed clinical and regulatory decisions can be made and treatment can be stopped or altered before organ injury occurs (Aronson 2005; Matheis et al. 2011; Sistare and DeGeorge 2011). The ideal characteristics of translational safety biomarkers of organ injury are that they provide more sensitive and specific information than current clinical chemistry biomarkers, can be detected through robust analytical assays in relevant translational species (rat, dog, mouse or monkey) and in humans, can be measured noninvasively or in accessible fluids like blood or urine, can predict or monitor severity of histopathology in nonclinical species, are specific for organ injury or mechanisms of toxicity that lead to organ injury, are specific to tissue location and are insensitive to nontoxic perturbations (exercise, diet, age, and other diseases and toxicities to other organs) (Amacher 2010; Muller and Dieterle 2009; Sasseville et al. 2014; Sistare and DeGeorge 2011; Mattes personal communication). The translational aspect, i.e., the ability of the biomarker to have similar responses in different species, enables comparisons of nonclinical studies with clinical studies. Figure 2 shows the process of discovering treatment-specific PD and omics biomarkers, followed by analytical verification and testing of the biomarkers in additional clinical and nonclinical studies for translational qualification. Biomarkers can be discovered in tissue or biofluids in nonclinical studies, but generally, the most useful biomarkers in the clinical setting are biomarkers detected in biofluids and are directly related to a mechanism of liver toxicity or a functional change in the liver. This review will focus on biomarkers of APAP-induced injury that are observed in urine and blood samples. Biomarkers may be related to the disposition and metabolism of the drug (such as APAP protein adducts) or to the endogenous response of the host to the drug. Endogenous phenotype indicators include changes in RNAs (e.g., mRNAs and miRNAs), proteins or metabolites; these can now be measured using the transcriptomics, proteomics and metabolomics platforms, collectively the omics technologies. The omics technologies have provided scientists with the ability to better characterize the phenotypes of patients and to discover biomarkers of diseases (Wood et al. 2014). When omics studies are applied to define an individual’s response to drugs, they are often referred to as pharmacogenomics, pharmacoproteomics and pharmacometabonomics or pharamacometabolomics, (Clayton et al. 2006; Everett et al. 2013; Hess 2013; Kaddurah-Daouk and Weinshilboum 2014; Lindpaintner 2002; Nicholson et al. 2011). In contrast, when the omics platforms are applied in toxicity studies, they are often referred to as toxicogenomics, toxicoproteomics and toxicometabolomics (Bouhifd et al. 2013; George et al. 2010; Guerreiro et al. 2003; Wetmore and Merrick 2004). There are several reviews of the omics technologies, the challenges associated with these technologies and how they can provide translational markers (Damia et al. 2011; Yang et al. 2012b).

The biomarker discovery phase usually involves collecting transcriptomics, proteomics and/or metabolomics datasets and using bioinformatics methods to process and analyze the data to discover biomarkers. Currently, the omics technologies are providing detailed phenotype information of a patient (Chen et al. 2012; Lobenhofer et al. 2008). Analysis of omics data usually involves multivariate statistical analyses such as principal component analysis (PCA), partial least squares discriminant analysis (PLS-DA) and other bioinformatics statistical methods (Kettaneh et al. 2005; Sánchez et al. 2012; Wold et al. 2001). Identification and selection of reproducible and translational biomarkers from omics datasets is a challenging problem for many reasons including small sample size, lack of quality control in sample collection and processing and failure to provide analytical verification of the biomarker, all of which have been addressed in other reviews (Broadhurst and Kell 2006; Ghosh and Poisson 2009; Poste 2012; Saeys et al. 2007). It is generally understood that new biomarkers discovered after application of bioinformatics analysis of omics datasets should be verified using more focused forms of analysis. The data acquired using multiple omics platforms may be evaluated individually in each omics dataset to discover biomarkers, or multiple omics datasets can be integrated to obtain a more comprehensive systems biology biomarker of the drug-induced hepatotoxicity (Ilyin et al. 2004). This approach allows systems biology biomarkers to be understood in terms of the molecular pathways affected by the toxicity (Laaksonen et al. 2006).

Transcriptomics examines the expression level of mRNAs in a given cell or tissue (Schena et al. 1998). Currently, isolated RNA can be used to evaluate both mRNA and microRNA (miRNA) (Lizarraga et al. 2012). MiRNAs are short (~22 nucleotide), single-stranded, noncoding RNA that regulate gene expression. Validation of transcriptomics biomarkers is usually accomplished by RT-qPCR to amplify the mRNA or miRNA to prove a specific RNA or DNA sequence was up- or down-regulated in the omics dataset (de Planell-Saguer and Rodicio 2011). This review will focus only on changes in miRNAs in relation to APAP-induced hepatotoxicity. Proteomics is the study of the proteome in a cell, tissue or biofluid (Anderson and Anderson 1998). Measuring the proteome in a sample can be done using several different techniques that include gel-based approaches such as 1D or 2D gels followed by liquid chromatography–mass spectrometry (Gel LC–MS) and gel-free approaches such as two-dimensional LC–tandem mass spectrometry (2D-LC–MS/MS). Validation of protein biomarkers is usually performed using immunohistochemical staining, enzyme-linked immunosorbent assay (ELISA) or western blot (Whiteaker et al. 2011). MS-based multiple reaction monitoring (MRM) techniques with peptide standards can be used in proteomics to quantify the amount of a potential protein biomarker in a sample (Becker et al. 2012; Gao et al. 2009). Metabolomics has been defined as “the measurement of the metabolite pool that exists within a cell under a particular set of conditions” (Fiehn 2002), while metabonomics was earlier defined as “the quantitative measurement of the dynamic multiparametric metabolic response of living systems to pathophysiological stimuli or genetic modification” (Nicholson et al. 1999). Metabolomics typically uses nuclear magnetic resonance (NMR) and/or MS to characterize the metabolites in a tissue or biofluid sample (Dunn et al. 2005). The advantages and disadvantages of NMR and MS in metabolomics have been discussed previously (Dunn and Ellis 2005; Robertson 2005). Metabolomics usually only reports the confidence of a chemical assignment to a peak and seldom reports analytical verification of a potential biomarker. Recently, several metabolomics groups have stated that MS/MS multiple reaction monitoring MRMs of reference standard compounds should be done for biomarker verification in LC/MS metabolomics studies (Kitteringham et al. 2009; Theodoridis et al. 2012). Finally, after an omics biomarker is discovered and analytically verified, the biomarker or pattern of biomarkers needs to be further evaluated in additional nonclinical and clinical studies to determine time domain, limitations of use and whether they are truly translational. It is best to test new biomarkers under multiple different scenarios to determine the domain of applicability of the biomarker (Altar et al. 2008; Matheis et al. 2011).

This review will cover biomarkers of APAP-induced liver injury that were discovered in omics nonclinical studies (Chen et al. 2008; Clayton et al. 2009; Coen et al. 2003; Reilly et al. 2001; Stamper et al. 2011; Sun et al. 2009; van Swelm et al. 2012; Yang et al. 2012b) or multiple omics nonclinical studies (Coen et al. 2004; Prot et al. 2011; Ruepp et al. 2002; Sun et al. 2012), whether these biomarkers went through analytical biomarker verification and whether these biomarkers have been observed in clinical studies (Bhattacharyya et al. 2014a, b; Fannin et al. 2010; Yang et al. 2015). This review will provide the status of APAP protein adducts and systems biology omics biomarkers (miRNAs, protein biomarkers and metabolite biomarkers) of APAP liver injury in the clinical setting. It will also discuss the role of statistics in discovery and validation of translational biomarkers, potential future applications of translational biomarkers of APAP liver injury and how these translational biomarkers can be used in the clinic to help make clinical decisions.

## Bioinformatics and statistics

With the rapid development of “omics” technologies, novel biomarker discovery in disease or toxicity is now mostly concerned with comprehensive analysis of potential biomarkers in biological samples such as cells, tissues or biofluids in a discovery-based or hypothesis-generating approach rather than the traditional hypothesis-based approach. Discovery-based approaches focus on identifying changes in relative abundances of new or novel molecular species that are statistically significantly associated with a disease or toxicity state. This type of study can often lead to subsequent investigations of the function of the candidate biomarker and hence is called hypothesis generating. Usually, the discovery-based approaches generate large, quantitative datasets of differentially expressed mRNAs, miRNAs, proteins or metabolites from primarily case–control experiments. Bioinformatics has a key role in facilitating the storage, analysis, dissemination and interpretation of such data. The basic workflow for analyzing such data involves: (1) data processing, (2) statistical analysis and validation and (3) high-level functional interpretation of the candidate biomarkers identified (Dunkler et al. 2011; Xia and Wishart 2011).

### Data processing and normalizations

Proper data cleansing/de-noising and normalizations of omics data are important processes in biomarker identifications. Large, high-throughput, machine-generated data often come with substantial numbers of missing values that need to be dealt with appropriately prior to statistical analyses. The presence of <1 % missing data points is generally considered trivial, and between 1 and 5 % is acceptable. However, when 5–15 % is missing, sophisticated methods to handle the dataset are required, and more than 15 % missing may severely impact any kind of interpretation (Acuna and Rodriguez 2004). Samples or features with too many missing values should be excluded while the rest can be imputed by appropriate computational methods that best suit the experimental data of interest, including replacement by a small value that is half of the minimum positive value in the input data table, mean or median, k-nearest neighbor, probabilistic principal component analysis and others. Outliers should be identified by boxplots, box-and-whisker plots or other methods and removed because many data analysis methods are sensitive to outliers. It is often necessary to normalize high-throughput data prior to statistical analysis in order to reduce systematic bias or technical variation or often to reduce the variance from more abundant features that dominate the variance–covariance matrix (Xia and Wishart 2011). Also, the classical statistical algorithms usually make the implicit assumption that the biomarkers follow a normal (Gaussian) distribution when in fact many omics datasets have heavily skewed distributions. In these cases, the data should be log-transformed in order to approximate the normal curve. There are excellent reviews available on normalizations of omics datasets including the paper by (van den Berg et al. 2006) for metabolomics data.

### Statistical analysis and validation

The main challenges associated with analysis of high-dimensional omics data for biomarker discovery are inherent bias in the data, small sample size relative to the large number of variables, excess false discovery rate due to multiple hypotheses testing and overfitting due to inadequate validation or cross-validation (Broadhurst and Kell 2006). The ‘short-and-wide’ structure of the data with the number of variables far exceeding the number of samples makes the data less conducive to the classical univariate statistical methods like *t*-tests because multiple independent hypotheses testing done in parallel across all the variables can result in a large number of false positives. The most common way to correct for high false-positive rates is to apply multiple testing corrections to the *p* values such as the false discovery rate (FDR) described by Benjamini–Hochberg (Benjamini and Hochberg 1995), the *q* value described by Storey and Tibshirani (Storey and Tibshirani 2003) or the more stringent Bonferroni correction (Dunn 1961). While these methods are routinely used in microarray-based studies, the downside to these methods is that it may result in loss of statistical power to detect the true positives. Also, univariate methods treat the individual variables as independent, which is largely not true in a biological system where a high degree of covariance is expected among the omics variables. As such, multivariate statistical methods that incorporate the covariance inherent in the omics data are increasingly being implemented (Wheelock and Wheelock 2013).

There are many multivariate algorithms that have been applied to omics data for biomarker discovery projects like factor analysis, linear discriminant analysis, canonical correlation analysis, multivariate ANOVA and artificial neural networks. The two most routinely used methods for exploratory analysis of omics data through dimension reduction are principal component analysis (PCA) and partial least squares discriminant analysis (PLS-DA). In general, multivariate methods can be broadly grouped into two categories: supervised methods and unsupervised methods. While the later uses no prior group identity to build the models, the former class of methods focuses on extracting the variables important to group separation. In PCA, an unsupervised multivariate method, the data are projected along transformed axes that represent orthogonal linear combinations of the original variables, thus maximizing the variance in the data. However, the challenge in PCA is to connect the observed group separation to the original variables used to build the PCA model. Therefore, PCA is used primarily as a first step in statistical modeling to assess data quality, detect outliers and provide a preliminary visual assessment of the strength of group separation in the data. PLS-DA, on the other hand, is a commonly used supervised multivariate method that performs multivariate correlation analysis between the predictor variables (the putative biomarker candidates) and the response variables (e.g., the group variable in case of case control studies). A major advantage of multivariate methods relative to univariate ones is that a single model is used to analyze all variables, and thus, the problems associated with multiple hypothesis testing are absent. However, the power of multivariate methods can be substantially diminished if the problem of overfitting is not dealt with appropriately (Wheelock and Wheelock 2013). Overfitting can result if a sufficient number of latent components are extracted such that the multivariate model results invariably in group separation that is convincing by visual inspection. Hence, evaluation of model parameters and model validation is of paramount importance in multivariate modeling. Typically, two parameters are assessed: the *R*^2^ value that indicates how well the model fits the data and the *Q*^2^ value that is the correlation based on averaging the results of multiple iterations of cross-validation. *Q*^2^ indicates the predictive power of the model. In general, *R*^2^ and *Q*^2^ values are expected to be close. If, however, *Q*^2^ is substantially lower than *R*^2^, the robustness of the model is poor implying overfitting. Overfitting can be reduced by determining the appropriate number of components, which is the cutoff point where *Q*^2^ starts decreasing with the addition of more components. Apart from cross-validation or related methods like bootstrapping and permutation tests, it is also highly recommended to use an independent dataset, the test set, to assess the accuracy of the model to eliminate model overfitting.

Variable selection is an essential step in multivariate methods for potential biomarker candidate detection and is usually done using the variable importance on the projection (VIP) parameter that summarizes the importance of each variable in deriving the group separation. A VIP score >1 is commonly used to select important variables besides assessing the loadings of each variable in the loadings plot. The variables with the highest VIPs are often evaluated in terms as discovered potential biomarkers.

Lastly, it is also worth mentioning that apart from reporting *R*^2^ and *Q*^2^ values as a measure of quality of a model, a receiver operating characteristic (ROC) plot of both training and overlaid test predictions is needed to claim utility of a particular predictive model. ROC curves plot the sensitivity of a predictive model against one minus the specificity for all possible values of the model threshold. The area under the curve (AUC) in a ROC plot summarizes the sensitivity/specificity trade-off of a predictive model over all possible thresholds. The AUC for random guessing is 0.5 (the diagonal in a ROC plot), while a perfect discrimination corresponds to an AUC of 1. ROC plots have been popularly used to assess the utility of biomarkers by comparing the AUC for predictive models with and without the biomarker (Alemayehu and Zou 2012).

## Clinical chemistry and APAP protein adducts

As mentioned above, the initiating events in APAP toxicity have been extensively reviewed (Jaeschke and Bajt 2006; James et al. 2003). Following a toxic dose of APAP, the conjugation pathways of the liver are overwhelmed and an increased proportion of APAP is metabolized by cytochrome (CYP) P450, predominantly CYP2E1, to the highly reactive metabolite NAPQI (Mitchell et al. 1973). While NAPQI is normally detoxified by glutathione, in APAP overdose, glutathione is depleted and NAPQI binds to cysteine on hepatic proteins as 3-(cystein-*S*-yl) APAP. Studies with antibodies that recognize total APAP protein adducts showed that adducts were specific biomarkers of APAP toxicity (Potter et al. 1989; Pumford et al. 1989, 1990; Roberts et al. 1987). The major antigenic determinant recognized by these antibodies consists of a cysteinyl sulfhydryl group (on a peptide or protein) covalently bound ortho- to the hydroxyl group and meta- to the acetamide on 3-(cystein-*S*-yl) APAP (Potter et al. 1989). Immunoassays performed in the mouse model of APAP toxicity determined the relationships between tissue adducts and toxicity, as measured by pathologic examination of the liver tissues and serum ALT levels (Potter et al. 1989; Roberts et al. 1991, 1987). It was shown that hepatocytes undergoing necrosis were the same cells that contained adducts and that the presence of adducts in liver cells preceded histological indication of necrosis and the elevation of serum ALT (Roberts et al. 1991). Immunoassays were also performed on blood samples of patients with acetaminophen liver injury and showed the presence of adducts in patients with high levels of ALT (James et al. 2001).

**Fig. 3:**
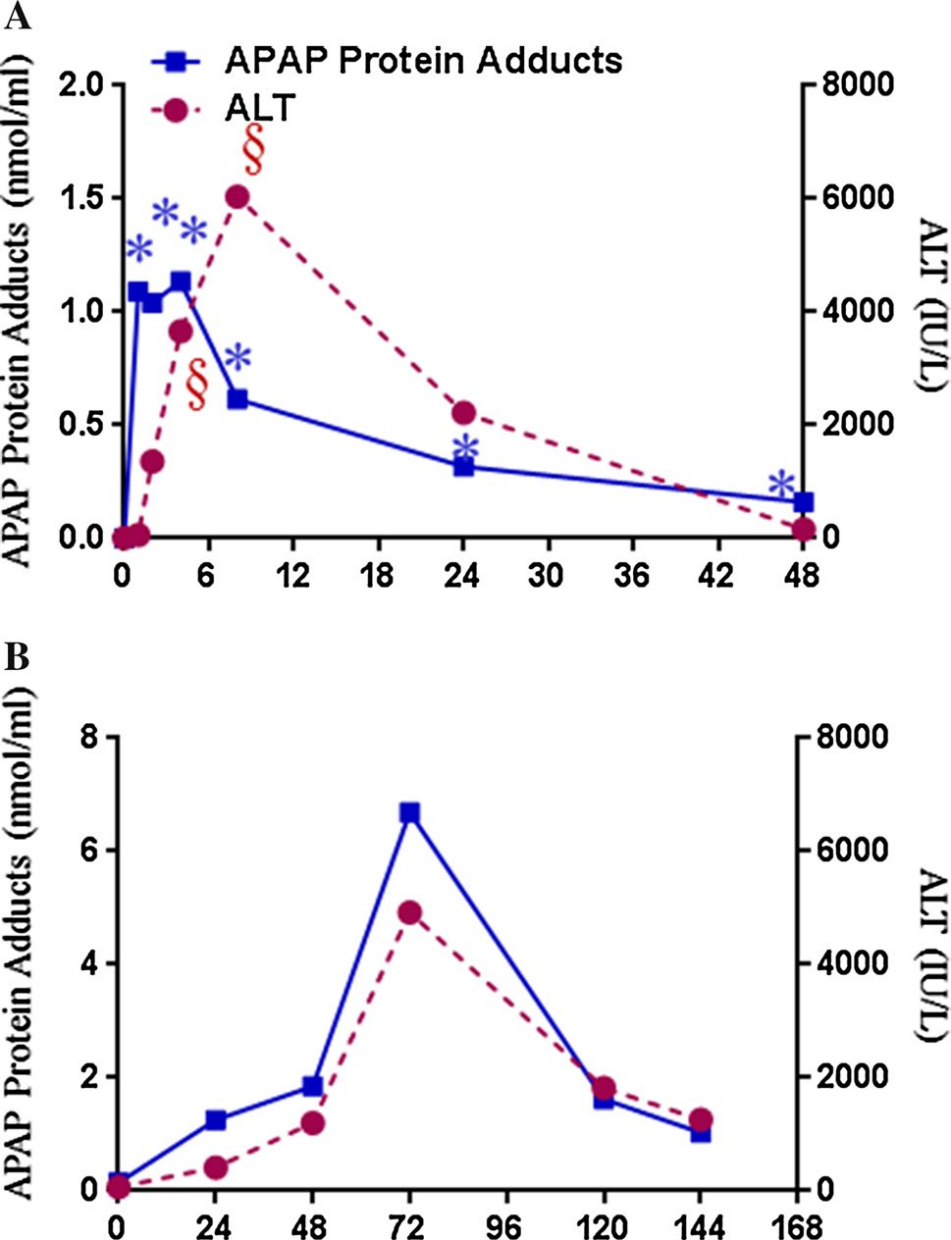
Plots showing time response of APAP protein adducts in **a** mice dosed with 200 mg/kg APAP (Bhattacharyya 2013) and **b** a human APAP overdose patient who was treated with *N*-acetyl cysteine (NAC) 31 h after APAP overdose (Bhattacharyya 2014b). APAP protein adducts and ALT data at 0 h reflect control values of non-APAP-treated mice (**a**), while APAP protein adducts and ALT values for **b** reflect control subjects in the clinical study

The development of a highly sensitive high-pressure liquid chromatography with electrochemical detection (HPLC–EC) adduct assay (Muldrew et al. 2002) allowed further study of APAP protein adducts in various clinical settings. Serum samples from patients with acute liver failure attributed to APAP overdose were found to have high levels of APAP protein adducts (Davern et al. 2006). Minimal levels of APAP protein adducts were detected in patients with ALF of known other etiology (e.g., viral hepatitis, autoimmune disease, Wilson’s disease, ischemia). In patients with ALF of unknown etiology (i.e., lack of a definitive diagnosis despite laboratory testing), up to 18 % of samples were shown to have toxic levels of adducts (Davern et al. 2006; Khandelwal et al. 2011). Moreover, the biochemical profiles (hyper-acute elevation of ALT, relatively low levels of bilirubin) and spontaneous recovery rates of these patients were consistent with those of the known APAP-induced ALF cases (Khandelwal et al. 2011). Thus, data generated by the HPLC-EC assay strongly implicated APAP as the etiology of the liver injury. ROC analysis of adduct levels in serum from patients known to have APAP hepatotoxicity showed that an APAP protein adduct level ≥1.1 nmol/mL had a high sensitivity and specificity (96.8 and 95.0 %, respectively) for patients with APAP overdose and ALT levels above 1000 IU/L. Thus, a level of 1.1 nmol/mL APAP protein adducts has been referred to as the “toxicity threshold level” (James et al. 2009). An analysis of the pharmacokinetics of adducts in 18 adults with APAP-induced liver injury reported an elimination half-life of 41.3 ± 8.3 h (James et al. 2009). By contrast, the elimination half-life of the parent drug APAP ranges from 2 to 18 h, depending on the severity of APAP liver injury (Bernal et al. 2010; Schiødt et al. 1997). Thus, the relatively long elimination half-life of adducts, an indicator of the oxidative metabolism of APAP, suggests that this biomarker could be useful in the clinical setting and has specificity for APAP-mediated liver injury. Figure 3a shows the time response of APAP protein adducts and ALT in mice after dosed with 200 mg/kg APAP (Bhattacharyya et al. 2013). APAP adducts increase rapidly before ALT and stay significantly increased at every time point in the study.

Additional studies have examined the quantitation of APAP protein adducts in patients receiving “therapeutic” or low-dose exposures of APAP. Adducts were quantified in the serum samples of healthy adults who participated in a study using a cross-over design to compare two formulations of APAP: the immediate release formulation and the sustained release formulation (James et al. 2013). Subjects received an 80 mg/kg dose of one APAP formulation, followed by a washout period and subsequent administration of the second 80 mg/kg formulation of APAP. The maximum plasma concentrations (C(max)) of adducts for immediate release (IR) and extended release (ER) formulations were 0.108 (±0.020) and 0.100 (±0.028) nmol/mL serum, respectively, and were two orders of magnitude lower than the maximum APAP adduct levels previously reported in adults with acute liver failure secondary to APAP. No changes in ALT levels were observed among the study participants. Another study compared the range of adduct levels in 24 healthy subjects receiving APAP daily doses of 4 g per day for a period of 10 days (Heard et al. 2011). The mean peak level of adducts for subjects in this study was 0.4 nmol/mL (±0.2 nmol/mL). Further study is needed to characterize adduct profiles in patients receiving low doses of APAP with preexisting disease (e.g., liver disease) to better understand the potential application of this biomarker across a range of APAP exposures. Overall, the data published on APAP protein adducts in human studies suggest that this biomarker is highly specific and sensitive for the detection of APAP liver injury. Figure 3b shows the time response of APAP protein adducts and ALT in a patient with late NAC treatment (31 h) (Bhattacharyya et al. 2014b). The time response of APAP adducts closely mirrored temporal changes in ALT for this patient.

## MiRNAs as APAP injury biomarkers

MiRNAs are short (~22 nucleotide), single-stranded, noncoding RNA that regulate gene expression posttranscriptionally by pairing with 3′ untranslated regions (3′-UTR) (Bartel 2004, 2009; Doench and Sharp 2004; Lewis et al. 2003; Turchinovich et al. 2011; Valadi et al. 2007; Wang et al. 2010). MiRNAs play important roles in basic cellular functions related to development, cellular differentiation, proliferation, apoptosis, cell-cycle control, metabolism and cancer (Iorio and Croce 2012). MiRNAs found in body fluids, including blood and urine, represent an important advance in biomarker discovery and may be useful noninvasive injury biomarkers in nonclinical and clinical studies. Most of these miRNAs are released into extracellular fluid via microvesicles, exosomes or protein-RNA complexes and are, therefore, highly stable (Yang et al. 2014). It has been suggested that miRNA may act not only within cells, but also in other tissues as extracellular signals (Cortez et al. 2011). Many miRNAs are expressed tissue-specifically or enriched in certain cell types. For example, miR-122 is one of the dominate miRNAs in the liver, accounting for 75 % of the total hepatic miRNA population (Wang et al. 2009; Lagos-Quintana et al. 2002), and has been identified as a APAP injury biomarker in animal (Wang et al. 2009) and clinical studies (Starkey Lewis et al. 2012). Table 1 lists miRNAs reported in animal and clinical studies and includes the biofluid used, discovery and verification and validation methods for the miRNA.

**Table 1:** Translational microRNA biomarkers of acetaminophen liver injury

microRNA biomarker	Nonclinical				Clinical		
	Gender and species	Biofluid	Discovery method	Verification method footnote for reference	Human subjects (# of control; # of APAP)	Biofluid (normalizer)	Validation method footnote for reference
miR-122	Male BALB/c mice	Plasma	Microarray	TaqMan^®^ qPCR^c^	25; 53	Plasma (U6 snRNA)	TaqMan^®^ qPCR^d^
miR-122	Female C57BL/6 mice	Serum/plasma	Protein-rich fraction	TaqMan^®^ qPCR^e^	0; 129	Plasma (let-7d)	TaqMan^®^ qPCR^f^
miR-122	Male Sprague–Dawley Rat	Serum	TaqMan^®^-based PCR array	TaqMan^®^ qPCR^g^	22; 22^b^	Plasma (cel-39 spike-in)	TaqMan^®^ qPCR^h^
miR-122^a^	Female C57BL/6 mice	Plasma	SYBR^®^ green-based PCR array	SYBR^®^ green-based PCR array^i^	12; 37	Serum/plasma (a set of 20 stable miRNAs)	SYBR^®^ green-based PCR array^j^
miR-122	Male Sprague–Dawley rat	Plasma	TaqMan^®^-based PCR array	TaqMan^®^ qPCR^k^	6; 6	Serum (miR-7i)	LNA™ based qPCR^l^
miR-122	Male Sprague–Dawley rat	Plasma	N/A	TaqMan^®^ qPCR^m^			
miR-192	Male BALB/c mice	Plasma	Microarray	TaqMan^®^ qPCR^c^	25; 53	Plasma (U6 snRNA)	TaqMan^®^ qPCR^d^
miR-192	Male Sprague–Dawley rat	Serum	N/A	TaqMan^®^ qPCR^g^	6; 6	Serum (miR-7i)	LNA™-based qPCR^l^
miR-192	Male Sprague–Dawley rat	Plasma	TaqMan^®^-based PCR array	TaqMan^®^ qPCR^k^			

^a^Plasma miR-122 down-regulation at the 12-h time point of this study

^b^22 subjects with ALT elevations exceeding 3 × baselines ALT compared to 22 matched patients without ALT

^c^(Wang et al. 2009)

^d^(Starkey Lewis et al. 2011)

^e^(Bala et al. 2012)

^f^(Antoine et al. 2013)

^g^(Su et al. 2012)

^h^(Thulin et al. 2014)

^i^(Ward et al. 2012)

^j^(Ward et al. 2014)

^k^(Yamaura et al. 2012)

^l^(Krauskopf et al. 2015)

^m^(Starckx et al. 2013)

### MiRNA profiling and data normalization

Compared to tissue samples, biofluids are difficult matrices to work with due to the limited amount of miRNAs. Real-time polymerase chain reaction (qPCR) and microarrays are the two well-established miRNA-profiling approaches for miRNA detection; next-generation sequencing (NGS) is another new method based on direct sequencing. The qPCR platform provides a larger dynamic range of miRNA detection and therefore requires less sample volume and provides more sensitive quantification. The advantages of NGS are its high-throughput detection of novel miRNAs and high accuracy in distinguishing miRNA isomers (Wang et al. 2014). Detailed discussions of the strengths and pitfalls can be found within a previous review (Pritchard et al. 2012).

Data normalization is a big challenge for biofluid miRNA analysis. Currently, there is no accepted standard to be used for miRNA normalization. Endogenous miRNAs have been proposed as powerful standards in terms of normalizing for some of the clinical variability. However, the levels of some commonly used normalizers (e.g., U6 snRNA) were different between control and disease subjects, because U6 expression profiles from young, aging and different disease conditions are variable (Qi et al. 2012). Exogenous spiked-in miRNAs were more consistent between samples; however, it only corrected the variations after the RNA purification. In the absence of a gold standard normalization method, it appears to be good practice to use multiple normalizers showing low variation within the samples.

### MiRNAs in animal models

**Fig. 4:**
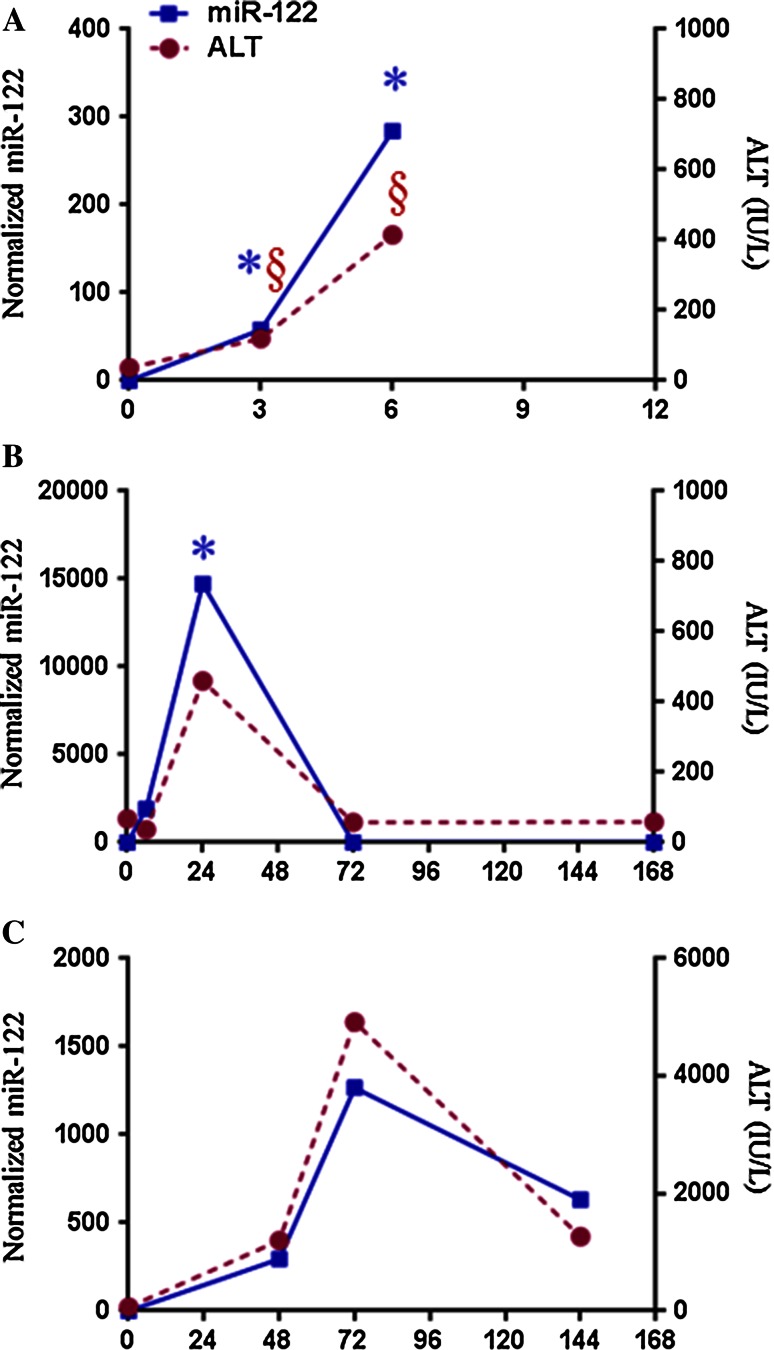
Plots showing time response of serum miR-122 versus ALT in **a** mice dosed with 500 mg/kg (approximation of Fig. 3b, c in Bala et al. 2012), **b** rats dosed with 1250 mg/kg APAP (Yang 2012a; unreported data) and **c** human APAP overdose patient (Yang et al. 2015). The miR-122 and ALT data shown at 0 h represent mean values of non-APAP-treated mice (**a**) or rats (**b**). Time 0 for figure **c** reflects mean values of parameters for non-APAP-treated children

Using the APAP-induced liver injury mouse model, the first study on circulating miRNAs as liver injury biomarkers was published in 2009 (Wang et al. 2009). Wang et al. (2009) reported that the level of many plasma miRNAs inversely correlated with the level of hepatic miRNAs, indicating that for these miRNAs, hepatic injury caused the release of the miRNAs into circulation. Specifically, miRNA-122 and miRNA-192 (Table 1), which are predominantly expressed in the liver, increased in the plasma with concurrent decreases in the liver. In addition, the increases in both miRNAs were detected earlier than the increase in ALT at 1 h post-treatment. At the later time points, 3 and 6 h after APAP, both plasma miR-122 and ALT started to increase, while the fold change of miR-122 is larger than of ALT (Fig. 4a; Bala et al. 2012). The peak values of ALT at different times (6, 24 and 72 h) are shown in Fig. 4a–c, and this is because dosing and genetic differences change the toxicokinetics in the nonclinical and human studies. Table 1 shows that several independent groups soon provided confirmatory and additional data supporting that miR-122 and miR-192 were elevated in APAP-induced hepatotoxicity. In APAP-overdosed mice (Table 1), another study confirmed the significant increase in miR-122 plasma and discovered three new potential biomarker miRNAs: miR-155, miR-146a and miR-125b (Bala et al. 2012). Moreover, this group identified 40 miRNAs in the plasma that were dysregulated among lethally and sub-lethally dosed mice (Ward et al. 2014). Significant alterations at 12 h with an APAP lethal dose were observed for ALT levels and 12 miRNAs: 574-5p, 135a*, 466 g, 1196, 466f-3p, 877, 342-3p, 195, 375, 29c, 148a and 652.

Similarly, the significant increase in miR-122 has been reported in rats (Starckx et al. 2013; Su et al. 2012; Yamaura et al. 2012), whose susceptibility to APAP-induced hepatotoxicity is less than that of the mouse, but is still a commonly used animal model. Using the miRNA qPCR array-based profiling approach, Su et al. (2012) found that serum miR-122, miR-192 and miR-193 levels were increased at 3 h while biochemical parameters [e.g., ALT and aspartate aminotransferase (AST)] remained at baseline in APAP-overdosed rats. However, both ALT and miR-122 showed peak elevation between 12 and 24 h after APAP overdose, and they returned to baseline at 3 days post-treatment in a similar pattern (Fig. 4b). In summary, the level of nonclinical circulating miR-122 appears to rise earlier than of ALT and then follow the response of the translational biomarker ALT.

Urine as a biofluid to evaluate miRNA biomarkers in APAP overdose situations has not been extensively utilized or directly compared to serum or plasma. Yang et al. (2012a) explored urinary miRNAs profiles for drug-induced liver injury in rat models. It was found that urinary miRNA profiles were altered in rats after administration of a toxic dose of APAP. The levels of 10 common urinary miRNAs (miR-185, miR-296, miR-20b-3p, miR-484, miR-330*, miR-434, miR-433, miR-34C*, miR-291a-5p and miR-664) were increased in both APAP-treated and carbon tetrachloride-treated rats (Yang et al. 2012a).

The above nonclinical studies laid the foundation to test miR-122 and miR-192 in clinical samples as stable diagnostic biomarkers in APAP overdose in humans (Table 1). Using the qPCR technique, Starkey Lewis and coworkers (Starkey Lewis et al. 2011) examined miR-122 and miR-192 in APAP-induced acute liver injury patients (*n* = 53) and showed that both miRNAs were elevated (Table 1). Both miRNAs are liver specific, and miR-122 is a marker of hepatocyte-specific differentiation and lipid metabolism. Starkey Lewis et al. (2011) reported that miR-122 correlated with ALT levels in the APAP cohort and had a much faster return to baseline than ALT. A recent study looked at circulating miRNAs by miRNA PCR array in patients with APAP-induced hepatotoxicity (*n* = 37) and ischemic hepatitis (*n* = 7) (Ward et al. 2014). APAP patients showed dramatic increase in some miRNAs (miR-122-5p, miR-27b-3p, miR-21-5p, miR-125b-5p, miR-194-5p, miR-193a-5p and miR-1290) before the ALT elevations above 1000 IU/L. A set of 11 miRNAs was shown to discriminate patients with APAP hepatotoxicity receiving the antidote NAC, from patients with ischemic hepatitis. A majority of the elevated miRNAs showed recovery with NAC treatment, but miR-1290 remained elevated for at least 2 days. This study was able to corroborate the previously reported elevations of miR-122, but failed to replicate the earlier report for miR-192 (Starkey Lewis et al. 2011). In addition, the validity of miR-122 as an APAP injury biomarker was supported in two recent investigations (Antoine et al. 2013; Thulin et al. 2014).

Recent developments in high-throughput NGS have enabled miRNA profiling in biofluids. A recent study using the NGS platform (Krauskopf et al. 2015) on a small number of samples (*n* = 6) from APAP-overdosed patients showed that 36 miRNAs were significantly enriched in the serum and returned to the baseline levels with NAC treatment. Significantly higher levels of miR-122, miR-483, and miR-23a were observed in APAP overdose subjects. This study was able to substantiate the findings of Starkey Lewis et al. (2011) for miR-122 and miR-192 in APAP overdose samples. The authors compared the miRNA data to a publicly available small RNA sequencing dataset across eight tissues (Faghihi et al. 2010) and found 12 miRNAs responsive to APAP overdose enriched in different tissues. Five miRNAs were enriched in liver: hsa-miR-122-5p, hsa-miR-192-5p, hsa-miR-483-5p, hsa-miR-194-5p and hsa-miR-210-3p, whereas other enriched tissue-specific miRNA were as follows: muscle-enriched miRNAs hsa-miR-193 b-5p, hsa-miR-378c and hsa-miR-378a-3p, frontal orbital gyrus-enriched miRNAs miR-125b-3p and hsa-miR-125b-5p, and pancreas-enriched miRNAs hsa-miR-148a-3p and hsa-miR-130b-3p. This enrichment analysis showed that besides liver injury, APAP also has effects on other organs such as muscle, pancreas and brain. A recent clinical study (Yang et al. 2015) using small RNA sequencing analysis identified eight serum miRNAs (miR-122, miR-375, miR-423-5p, miR-30d-5p, miR-125b-5p, miR-4732-5p, miR-204-5p and miR-574-3p) which were increased more than twofold in APAP overdose samples (*n* = 8). Interestingly, the time-dependent change in serum miR-122 is similar to the ALT change (Fig. 4c) and shows in this APAP overdose patient the elevated serum miR-122 levels returned to baseline before serum ALT. Yang et al. (2015) also made use of the urine as biofluid and detected in APAP overdose samples significantly increased levels of miR-375, miR-940, miR-9-3p and miR-302a. These different miRNA expressions suggest specific functional significance in fine-tuning the target mRNA levels at various stages of the APAP toxicity. When using these three high-throughput platforms (Krauskopf et al. 2015; Ward et al. 2014; Yang et al. 2015), the common miRNAs in APAP overdose patients were miR-122 and miR-125b-5p. Because of very short complementarity to the target mRNA, individual miRNAs can repress large sets of mRNAs (Giraldez et al. 2005; Lim et al. 2005) primarily through interaction with 3′-UTR. For example, studies with miR-122 has also shown it as key regulator of multiple pathways involved in sepsis and coagulation (Wang et al. 2014), inflammatory diseases (Roderburg et al. 2015), hepatocarcinogenesis (Hsu et al. 2012; Tsai et al. 2012) and lipid metabolism (Esau et al. 2006). Our review of the miR-122 in APAP overdose clinical studies highlights the challenges inherent in comparing miRNA abundance in different clinical samples. The relationship between miRNA-122 elevation and mechanisms of APAP toxicity remains unclear. Thus, future studies are needed in this area, as well as better characterization of kinetics and elucidation of target mRNAs.

In summary, recent studies have shown the presence of miRNAs in biofluids and their potential as biomarkers of organ injury.

## Proteomics APAP injury biomarkers

Proteomics and toxicoproteomics methods have been used to discover biomarkers of acetaminophen liver injury. The first studies focused on biomarkers in liver tissue, but recent studies have begun looking for injury biomarkers in urine and blood samples. The field of proteomics has benefitted from rapid increases in analytical and computational capabilities since the field was initiated in 1992 and, at the time, relied primarily on large 2D gels to separate the proteins. The different analytical methods currently used in clinical proteomics have been detailed (Boja et al. 2010; Frantzi et al. 2014). Generally, there is an untargeted discovery process involving control and dosed samples from nonclinical or clinical studies (Amacher 2010). Since it is hard to obtain clinical samples in overdose cases that control the many genetic and environmental variables that can affect protein expression, it is often easier to discover proteomics biomarkers in nonclinical toxicity studies and then set up clinical sample collection strategies to further evaluate the potential toxicity biomarkers. Another factor for proteomics is that it is of relatively low throughput compared to other omics technologies in all three phases of discovery, verification and validation and, therefore, fewer biomarkers have been discovered and validated in proteomics. The two major untargeted approaches in proteomics discovery are gel-based and gel-free methods to identify differentially expressed proteins in biological samples (Boja et al. 2010). There are many mass spectrometry-based proteomics analytical methods (Boja et al. 2010) that include matrix-assisted laser desorption/ionization time of flight (MALDI-Tof), surface-enhanced laser desorption/ionization time of flight (SELDI–Tof) and electrospray ionization liquid chromatography tandem mass spectrometry (ESI LC–MS/MS). There are several chemical labeling methods including isotope-coded affinity tag (ICAT), isobaric tags for relative and absolute quantitation (iTRAQ), mass differential tags for relative and absolute quantification (mTRAQ) and stable isotope labeling by amino acids in cell culture (SILAC) to aid the analytical process. Other proteomics analytical platforms include flow cytometry and enzyme-linked immunosorbent assay (ELISA). For quicker results, protein arrays such as forward-phase protein array (FPPA) using antibodies, reverse-phase protein array (RPPA) that measures only protein with one antibody but multiple samples and nucleic acid-programmable protein array (NAPPA) are used. There is a big push for quantitative proteomics methods that will make it easier to make interlaboratory comparisons (Abdallah et al. 2012; Gao et al. 2009). The identified proteins are then statistically analyzed for statistical significance and used in pattern recognition algorithms to identify a pattern of proteins associated with the disease or drug effect. Often, the proteins with the highest potential to be biomarkers are then verified using other analytical techniques such as multiple reaction monitoring (MRM) using a triple quadrupole (TQ) MS, western blots or ELISAs. Additional studies are needed to validate the biomarkers in a clinical setting; this is often done by ELISA or MRM (Marx 2013).

Table 2 lists 11 potential protein translational biomarkers of acetaminophen-induced liver injury. Three of the biomarkers were found in urine while the remaining 8 were found in serum or plasma. The table lists the biomarkers followed by four columns with relevant nonclinical information about the discovery process, and the last three columns have relevant information about the current status of clinical validation of the potential protein biomarker of liver injury. The first three protein biomarkers in Table 2 were identified in an APAP study with male FVB mice using MALDI-Tof MS open profiling and MALDI linear ion trap MS for peptide and protein identification (van Swelm et al. 2012). Analysis of the MALDI data resulted in 12 differentially expressed proteins that correlated with liver injury as measured by ALT. Three of the proteins, superoxide dismutase 1 (SOD1), calmodulin (CaM) and carbonic anhydrase (CA3), were then analytically verified using western blots. CaM was reported to increase before ALT was increased in blood. Evaluation of these three biomarkers was performed in urine samples from 24 control patients, one severe APAP overdose patient and 10 additional patients suspected of drug-induced acute liver injury (ALI) where eight of the 10 ALI patients were linked to APAP ingestion. Western blot analysis of SOD1 and CA3 showed that these proteins were present in APAP overdose patients and were not observed in the pooled control sample. ELISA analysis of CaM showed that it increased in samples from subjects with liver injury and correlated with increasing APAP concentration; CaM also increased prior to ALT increase. SOD1 has been previously reported as a biomarker of liver injury (Agarwal et al. 2012; Smyth et al. 2008). CaM plays a role in maintaining Ca^2+^ balance which can be disrupted when NAPQI binds to mitochondrial proteins (Ray et al. 1990). High levels of Ca^2+^ are brought to the nucleus by CaM where it will cause DNA fragmentation and lead to necrosis (Nicotera et al. 1989). Limitations of these studies are that they were observed in urine where most urinary proteins are from the kidney, the multitude of dilution factors that can affect the concentration of urinary markers and only one time point (24 h) was evaluated. Therefore, additional studies are needed to further evaluate the potential of urinary SOD1, CA3 and CaM as sensitive and specific markers of liver injury biomarkers due to acetaminophen and other causes of liver injury.

**Table 2:** Translational protein biomarkers of acetaminophen liver injury

Protein biomarker	Nonclinical				Clinical		
	Gender and species	Biofluid	Discovery method	Analytical verification method footnote for reference	# of control; # of APAP patients	Biofluid	Analytical Validation method footnote for reference
Superoxide dismutase 1 (SOD1)	Male FVB mice	Urine	MALDI-Tof MS	Western blot^c^	24; 11	Urine	Western blot^c^
Calmodulin (CaM)	Male FVB mice	Urine	MALDI-Tof MS	Western blot^c^	24; 11	Urine	Western blot and ELISA^c^
Carbonic anhydrase 3 (CA3)	Male FVB mice	Urine	MALDI-Tof MS	Western blot^c^	24; 11	Urine	Western blot^c^
Keratin-18 (FL-K18) and (cK18)	Male CD-1 mice	Serum	Gel LC MS/MS^d^		31; 84^a^	Serum or Plasma	LC MS/MS and ELISA^e^
High-mobility group box-1 (HMGB1)	Male CD-1 mice	Serum	Gel LCMS/MS^f^		31; 84^a^	Serum or Plasma	LC MS/MS and ELISA^e^
Argininosuccinate synthetase (AS)	Male C57 Bl6 mice	Plasma	Gel LC MS/MS^f^	ELISA^g^	6; 21^b^	Plasma	ELISA^g^
Betaine–homocysteine *S*-methyltrans (BHMT)	Male C57 Bl6 mice	Plasma	Antibody and/or iTRAQ LC–MS/MS	Western blot^h^	4; 4	Plasma	Western blot^h^
Fumarylacetoacetate hydrolase (FAH)	Male C57 Bl6 mice	Plasma	Antibody and/or iTRAQ LC–MS/MS	Western blot^h^	4; 4	Plasma	Western blot^h^
Fructose-1,6-bisphosphatase 1 (FBPI)	Male C57 Bl6 mice	Plasma	Antibody and/or iTRAQ LC–MS/MS	Western blot^h^	4; 4	Plasma	Western blot^h^
Dihydropyrimidinase (DPYS)	Male C57 Bl6 mice	Plasma	Antibody and/or iTRAQ LC–MS/MS	Western blot^h^	4; 4	Plasma	Western blot^h^
Hydroxyphenyl-pyruvate dioxygenase (HPD)	Male C57 Bl6 mice	Plasma	Antibody and/or iTRAQ LC–MS/MS	Western blot^h^	4; 4	Plasma	Western blot^h^

^a^78 APAP high ALT and six APAP normal ALT

^b^13 APAP high ALT and nine APAP normal ALT

^c^(van Swelm et al. 2012)

^d^(Antoine et al. 2009)

^e^(Antoine et al. 2012)

^f^(Svetlov et al. 2006)

^g^(McGill et al. 2014a, b)

^h^(Hu et al. 2014)

Keratins are responsible for cell structure and integrity. Full-length keratin-18 (FL-K18) and caspase-cleaved keratin-18 (cK18) are necrosis and apoptosis markers, respectively (Caulín et al. 1997; Schutte et al. 2004), that are released into the blood. The high-mobility group box-1 (HMGB1) protein is a pro-inflammatory nuclear protein that is passively released and targets Toll-like receptors (TLR) to alert the immune system of dying cells (Lotze and Tracey 2005; Scaffidi et al. 2002). The analysis of HMGB1 and K18 by gel LC/MS/MS in a nonclinical study in mice showed that they both increased significantly at early time points after APAP exposure (Antoine et al. 2009). A clinical study of HMGB1 and K18 measured by ELISA and immunoblot (Antoine et al. 2012) found that FL-K18 and cK18 were highly correlated with ALT for up to 24 h after APAP exposure. ROC scores of 0.90 for FL-K18, 0.84 for cK18 and 0.87 for HMGB1 were all higher than the ROC score for ALT (0.80) in the nonclinical study (Antoine et al. 2009). The clinical study showed that K18 and HMGB1 are translational blood protein markers of necrosis and immune response in acetaminophen overdose patients (Antoine et al. 2012). Additional studies are needed to further determine the limitations of the translational protein markers in other conditions such as liver disease during APAP-induced liver injury.

Argininosuccinate synthetase (AS) represents a potential translational protein liver injury biomarker that was initially observed by Svetlov et al. (2006) as a biomarker of liver perfusion injury and then further evaluated in nonclinical and clinical APAP overdose studies by McGill et al. (2014a). AS catalyzes the formation of argininosuccinate from citrulline and aspartate and is degraded in the liver during liver injury and released into the circulation. AS was identified using immunoblotting and cation–anion exchange chromatography/reversed-phase liquid chromatography–tandem mass spectrometry (Svetlov et al. 2006). In mice, AS increased fivefold at 2 h after 300 mg/kg APAP exposure, prior to the elevation of ALT at 6 h. AS was then evaluated by ELISA in APAP overdose patients by ELISA (McGill et al. 2014a). Thirteen APAP overdose patients with liver function tests [ALT > 1000 U/L and evidence of coagulopathy as indicated by a prothrombin time (PT > 18 s)] were classified as “abnormal LT” while nine APAP overdose patients who had liver test (peak ALT < 100 U/L and PT < 18 s) were classified as “normal LT.” There were six healthy control patients in the study. There was a 5000-fold increase in AS versus a 156-fold increase in ALT in humans with abnormal LT. The increase in humans was a bigger response than the increase observed in mice. AS increased more rapidly than ALT and decreased more rapidly than ALT in both mice and humans, making it a potential translational biomarker of liver injury. More studies are needed to determine specificity of AS and how much earlier AS increases before ALT in nonclinical and clinical studies.

The last five potential translational protein biomarkers of liver injury listed in Table 2 were recently reported (Hu et al. 2014) using multiple proteomics technologies. This study used label-free antibody array surface plasmon resonance technology, targeted iTRAQ MS and quantitative western blots to discover 20 potential protein biomarkers of liver injury in a nonclinical study of APAP-dosed mice using two APAP doses and seven time points. Membrane-bound catechol-O-methyltransferase (MB-COMT) and retinol binding protein 4 (RBP4) were two protein liver injury markers that were altered before ALT changes were observed in the nonclinical study. Quantitative western blots were used to monitor the potential protein liver injury biomarkers in a small clinical population of four control patients and four APAP overdose patients. Five of the 20 proteins discovered in the nonclinical study were observed by western blots in the clinical study. The five proteins observed in the clinical study were betaine–homocysteine *S*-methyltransferase 1 (BHMT), fumarylacetoacetate hydrolase (FAH), fructose-1,6-bisphosphatase 1 (FBP1), dihydropyrimidinase (DPYS) and 4-hydroxyphenylpyruvate dioxygenase (HPD). BHMT is involved in oxidative stress, FBPI is involved in glycolysis, while FAH and HPD are involved in tyrosine catabolism pathway, and these are known to be involved in APAP liver injury. It is noteworthy that all five proteins observed in the clinical study are located in the cytoplasm and these proteins may be more abundant than proteins at other cellular locations that were not observed in the clinical study. Also, in this study BHMT, FAH, FBP1 and HPD were shown to be primarily liver-specific proteins while DPYS was liver enriched and observed in liver and kidney. BHMT, FAH, FBP1, DPYS and HPD need to be evaluated in larger clinical APAP overdose and acute liver injury studies using other protein detection methods besides western blots to better determine their sensitivity and specificity before they can be validated as translational and mechanistic biomarkers of liver injury.

## Metabolomics for evaluation of APAP injury biomarkers

The liver is the major organ for the synthesis of endogenous compounds and for the metabolism of exogenous compounds. Therefore, it is reasonable to hypothesize that the introduction of exogenous compounds to the biosystem would alter the endogenous metabolic profile in the liver and in biofluids and that these changes could occur prior to changes in standard clinical parameters such as ALT and precede overt liver injury (O’Connell and Watkins 2010). Changes in metabolite biomarkers are expected to be observed earlier than those liver injury protein biomarkers because proteins/enzymes are only released from dead cells after injury occurs (McGill et al. 2012). From APAP nonclinical studies, metabolic biomarkers including those related to oxidative stress, mitochondrial function and liver function have been reported with these changes occurring at early time points after dosing and prior to overt liver injury (Bhushan et al. 2013; Chen et al. 2008, 2009; Coen et al. 2003; Sun et al. 2008, 2009). Among these proposed metabolite biomarkers, few have been further tested in clinical studies (Table 3). In this section, the focus will be on acylcarnitines, bile acids and pyroglutamic acid, which have been evaluated in both rodents and humans as potential markers related to APAP-induced liver injury.

**Table 3:** Translational metabolite biomarkers of acetaminophen liver injury

Metabolite biomarkers	Nonclinical				Clinical		
	Gender and species	Biofluid	Discovery method	Verification method (ref)	Human subjects	Biofluid	Validation method (ref)
Palmitoyl carnitine Myristoyl carnitine Oleoyl carnitine	Male Sprague–Dawley rats	Serum	LC/QTof–MS	LC/TQ–MS^e^	23; 187; 62^a^	Serum	UPLC–TQ/MS^h^
	Female wild-type (Cyp2e1 +/+) and Cyp2e1-null mice; male wild-type (Pparα +/+) and Pparα-null mice	Serum	LC/QTof–MS	LC/QTof–MS^f^			
	Male B6C3Fone mice	Serum	LC/QTof–MS	LC/TQ–MS^g^			
Palmitoyl carnitine Oleoyl carnitine Linoleoylcarnitine	Male C57Bl/six mice	Serum	LC/QTof–MS	LC/QTof–MS^i^	6; 14; 16^b^	Plasma	LC/QTof–MS^i^
Pyroglutamic acid	Male Sprague–Dawley rats	Serum	LC/QTof–MS	LC/QTof–MS^e^	One female patient with chronic APAP use	Urine	GC/MS^j^
					4	Urine/Plasma	GC/MS^k^
Cholic acid	Male Sprague–Dawley rats	Serum	LC/QTof–MS	LC/TQ–MS; LC/MS/MS^e^	19; 15; 64^a^	Serum	UPLC/TQ–MS^o^
	Male Sprague–Dawley rats	Urine	UPLC/Tof–MS	GC/MS^l^			
	Male Sprague–Dawley rats	Serum		LC/MS/MS^m^			
	Male Crl:CD (SD) rats	Urine/plasma		UHPLC–MS/MS^n^			
Deoxycholic acid	Male Sprague–Dawley rats	Serum	LC/QTof–MS	LC/TQ–MS^e^	19; 15; 64^a^	Serum	UPLC/TQ–MS^o^
	Male Sprague–Dawley rats	Urine	UPLC/Tof–MS	GC/MS^l^			
Glycochenodeoxycholic acid	Male Sprague–Dawley rats	Serum		LC/TQ–MS^e^	19; 15; 64^a^	Serum	UPLC/TQ–MS^o^
	Male Crl:CD (SD) rats	Plasma		UHPLC/MS/MS^n^			
Glycodeoxycholic acid	Male Crl:CD (SD) rats	Plasma		UHPLC/MS/MS^n^	19; 15; 64^a^	Serum	UPLC/TQ–MS^o^
					6; 9–12^c^	Plasma	UPLC/QTof–MS^p^
					31; 31^d^	Serum	UPLC/QTof–MS^p^
Glycocholic acid	Male Sprague–Dawley rats	Serum	LC/QTof–MS	LC/TQ–MS; LC/MS/MS^e^	19; 15; 64^a^	Serum	UPLC/TQ–MS^o^
	Male Sprague–Dawley rats	Serum		LC/MS/MS^m^	6; 9–12^c^	Plasma	UPLC/QTof–MS^p^
	Male Crl:CD (SD) rats	Plasma		UHPLC/MS/MS^n^	31; 31^d^	Serum	UPLC/QTof–MS^p^
Taurochnodeoxycholic acid	Male Sprague–Dawley rats	Serum		LC/QTof–MS^e^	19; 15; 64^a^	Serum	UPLC/TQ–MS^o^
	Male Crl:CD (SD) rats	Plasma		UHPLC/MS/MS^n^	6; 9–12^c^	Plasma	UPLC/QTof–MS^p^
					31; 31^d^	Serum	UPLC/QTof–MS^p^
Taurochloric acid	Male Sprague–Dawley rats	Serum	LC/QTof–MS	LC/TQ–MS; LC/MS/MS^e^	19; 15; 64^a^	Serum	UPLC/TQ–MS^o^
	Male Sprague–Dawley rats	Serum		LC/MS/MS^m^	6; 9–12^c^	Plasma	UPLC/QTof–MS^p^
	Male Crl:CD (SD) rats	Plasma		UHPLC/MS/MS^n^	31; 31^d^	Serum	UPLC/QTof–MS^p^

^a^Control; therapeutic dose; overdose

^b^Control; normal liver test result overdose group; abnormal liver test result overdose group

^c^Control; APAP-induced acute liver injury

^d^APAP-induced acute liver failure, survivor; APAP-induced acute liver failure, nonsurvivor

^e^Sun et al. (2013)

^f^Chen et al. (2008)

^g^Bhattacharyya et al. (2013)

^h^Bhattacharyya et al. (2014a, b)

^i^McGill et al. (2014a, b)

^j^Duewall et al. (2010)

^k^Fenves et al. (2006)

^l^Kumar et al. (2012)

^m^Luo et al. (2014)

^n^Yamazaki et al. (2013)

^o^James et al. (submitted)

^p^Woolbright et al. (2014)

### Acylcarnitines as biomarkers of APAP-induced liver injury

**Fig. 5:**
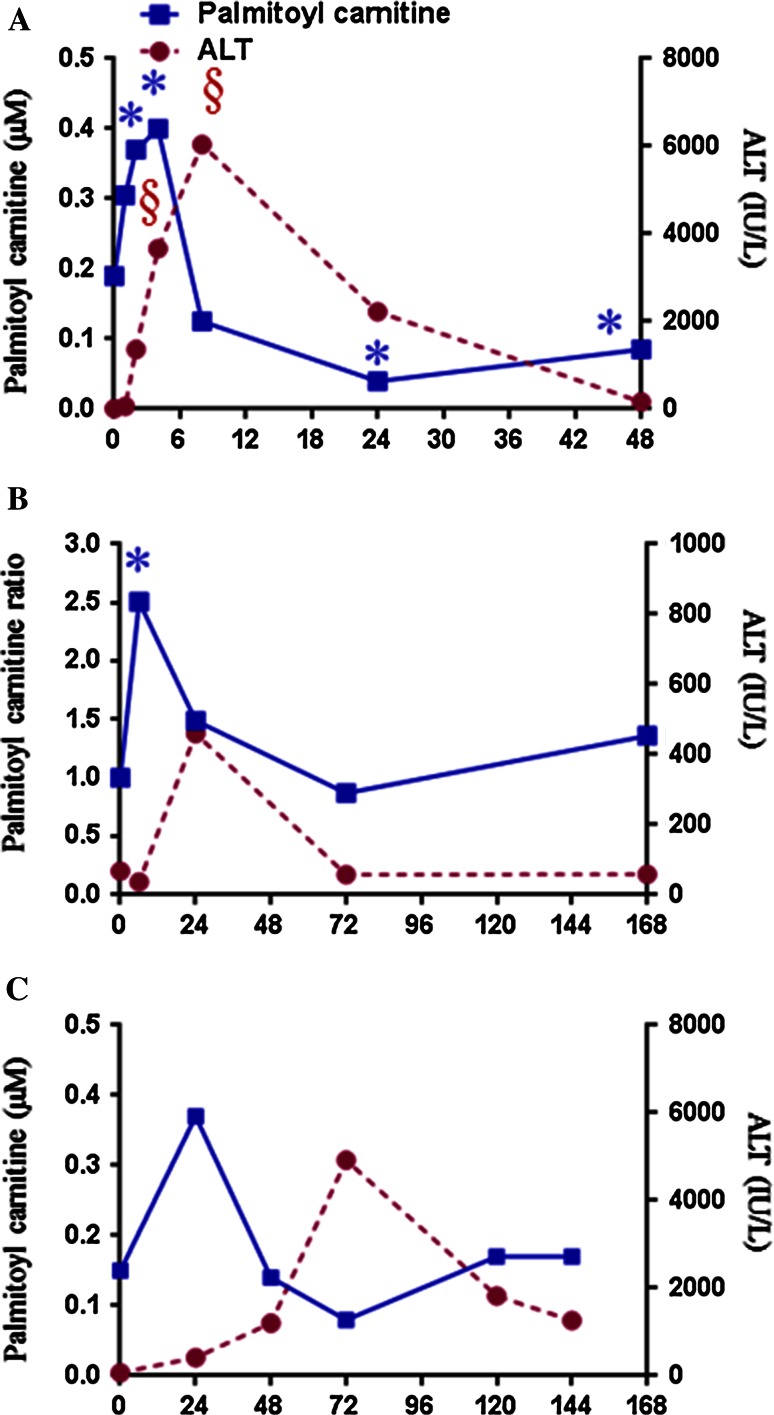
Plots showing time response of blood palmitoyl carnitine versus ALT in **a** mice dosed with 200 mg/kg (Bhattacharyya et al. 2013), **b** rats dosed with 1250 mg/kg APAP (Sun et al. 2013) and **c** human APAP overdose patient (Bhattacharyya et al. 2014a). Palmitoyl and ALT data at 0 h are mean values of non-APAP-treated mice or rats. Time 0 for the patient reflects mean values for non-APAP-treated children from the clinical study

Acylcarnitines are intermediate forms of fatty acids that can be transported into the mitochondria for fatty acid β-oxidation. Fatty acid β-oxidation involves three reactions: (1) activation of fatty acids to long-chain fatty acyl-CoA catalyzed by acyl-CoA synthetase in the cytosol; (2) transport into mitochondria after conversion of the fatty acyl-CoA to acylcarnitines catalyzed by carnitine acyltransferase I in the inner mitochondria membrane; and (3) degradation to acetyl CoA catalyzed by enzymes in the mitochondrial matrix. APAP has been reported to cause mitochondrial dysfunction (Kon et al. 2004) and disruption of energy metabolism (Chen et al. 2009; Coen et al. 2003) with resulting accumulation of blood levels of acylcarnitines. The mitochondrial damage will result in acylcarnitine accumulation in blood. Chen et al. (2009) utilized LC/MS-based metabolomics to profile serum samples from control, APAP-treated wild-type and Cyp2e-1-null mice. They reported accumulations of long-chain acylcarnitines, triglycerides and free fatty acids in the serum of APAP-treated wild-type mice (compared with control) after APAP treatment, consistent with disruption of fatty acid oxidation. Palmitoyl carnitine was also increased in the wild-type mice and slightly lower in the Cyp2e1-null mice. Recently, Bhattacharyya et al. (2013) found increased levels of acyl carnitines in sera, which is consistent with disruption of fatty acid oxidation. Palmitoyl carnitine was also increased in the wild-type mice and slightly lower in the Cyp2e1-null mice. Figure 5a shows the concentration of palmitoyl carnitine and level of ALT at multiple time points after exposure of mice to APAP. Similar to the rat study, palmitoyl carnitine was at a maximum prior to the maximum of ALT, indicating that palmitoyl may represent an early marker of APAP-induced hepatotoxicity. Similar results were observed in a previous study (Sun et al. 2013) listed in Table 3. In this study, both open metabolomics profiling and broad metabolic profiling were employed to evaluate metabolome changes in serum from rats dosed with 100 mg APAP/kg body weight or 1250 mg APAP/kg body weight. Palmitoyl carnitine and fatty acid levels (including palmitic acid, palmitoleic acid, stearic acid, oleic acid, arachidonic acid and docosahexaenoic acid) were increased at 6 h post-dosing with 1250 mg APAP/kg body weight (Sun et al. 2013). Figure 5b shows the plots of the ratio of palmitoyl carnitine in APAP-treated rats to control and the corresponding ALT levels over four time points. The plots indicate that palmitoyl carnitine is at a maximum value prior to the maximum of ALT.

To further evaluate the use of acylcarnitines as clinically relevant markers of APAP-induced hepatotoxicity, several studies have been initiated to measure acylcarnitines levels in human sera (Bhattacharyya et al. 2014b; Fannin et al. 2010; McGill et al. 2014b). In one clinical study (Bhattacharyya et al. 2014b), the levels of long-chain acylcarnitines (palmitoyl, myristoyl and oleoyl carnitines) were measured in serum collected from three groups of children as follows: children receiving therapeutic doses of APAP (*n* = 187), children hospitalized for an APAP overdose (*n* = 62) and children without APAP exposure in the preceding 14 days (controls; *n* = 23). Significant increases in long-chain acylcarnitines (palmitoyl, myristoyl and oleoyl carnitine) were observed in subjects with APAP exposure compared with control, but no differences between the therapeutic and overdose group were noted. Figure 5c shows the concentration plots of palmitoyl carnitine and ALT levels over multiple time points in a single patient. Palmitoyl carnitine shows the same trend noted in the rodent studies with palmitoyl carnitine elevated before the maximum of ALT is achieved. The mouse, rat and human data indicate the potential of palmitoyl carnitine to serve as a translational biomarker of APAP-induced hepatotoxicity. Significant increases in serum long-chain acylcarnitines were observed in overdose subjects who received delayed (>24 h after overdose) treatment with the antidote NAC. The lack of elevation of serum levels of acylcarnitines in some patients with APAP overdose was likely due to early treatment with the antidote NAC. McGill et al. (2014) provided further evidence that the serum acylcarnitines levels (Table 3) were influenced by antidote treatment in patients. This report also examined acylcarnitines levels in APAP-treated mice that received NAC. Elevations of acylcarnitines were blunted in mice that received NAC 1.5 h after APAP, compared to mice that did not receive NAC. No significant increases in plasma acylcarnitine levels were observed in patients with APAP overdose after NAC treatment. Therefore, the acylcarnitines as biomarkers alone are not sufficient to predict APAP-induced hepatotoxicity, but can serve as complimentary biomarkers in combination with the current clinical markers. They may also serve as indicators for mitochondrial dysfunction if NAC or another antidote is not provided to the patient after overdose.

### Bile acids as biomarkers of APAP-induced liver injury

Bile acids (BAs) are important molecules for many processes in the liver and gastrointestinal tract including maintenance of energy homeostasis, activation of nuclear receptors and cell signaling pathways, cell proliferation and inflammation (Hylemon et al. 2009; Trauner et al. 2010). While the BAs have many important roles, they can also cause apoptosis, necrosis and oxidative stress (Allen et al. 2011; Copple et al. 2010; Fang et al. 2004; Gupta et al. 2004; Jaeschke et al. 2002). They have also been shown to be involved in the stimulation of liver regeneration (Borude et al. 2012; Chen et al. 2011; Meng et al. 2010). The primary bile acids are synthesized in the hepatocytes. Circulation of the BAs is highly dependent on transporting from the hepatocytes into biliary tracts, a process that is highly susceptible to perturbation from even minor liver damage indicating that they may be sensitive biomarkers of liver injury (Yamazaki et al. 2013). Total BAs were first noted to be altered in APAP toxicity in the clinical setting in the 1970s and were noted to be more sensitive than the clinical measures at that time (Hamlyn et al. 1977; James et al. 1975; Kaplowitz et al. 1973; Korman et al. 1974). Generally, the clinical measurement of BAs is of total BAs, which represent over 20 BAs in most species (Luo et al. 2014). It has been proposed that the measurement of individual BAs may be more predictive of the type of liver injury (Alnouti et al. 2008; Bentayeb et al. 2008; Ducroq et al. 2010; Turley and Dietschy 1978). Recent nonclinical and clinical studies indicate that the bile acids may play a critical role in liver injury and regeneration in response to APAP and may prove useful as biomarkers of these events in a clinical setting.

In a study designed to evaluate the role of bile acids in APAP overdose, C57BL/6 mice were divided into three groups and fed different diets for 1 week, after which time the mice received a single dose of APAP (Bhushan et al. 2013). Mice were killed at multiple time points up to 24 h after APAP treatment. An enzymatic method was used to evaluate total bile acids in the serum and liver tissue while a targeted UPLC/MS method was also employed to evaluate select hepatic bile acids. The results indicated that in mice on the diet supplemented with cholestyramine, which depletes bile acids, APAP toxicity was exacerbated, whereas those on a diet supplemented with cholic acid had a more rapid recovery from APAP. While this study did not seek to identify biomarkers related to injury progression and recovery, it is important in that it demonstrated the critical role of bile acids in APAP-induced liver injury. A UPLC/MS-based metabolomics study was employed to evaluate urinary biomarkers of hepatotoxicity in rats dosed with known hepatotoxins including APAP (Kumar et al. 2012). APAP was administered for five consecutive days, and urine collected at 24 and 48 h after the last dose. Compared to the control mice, urinary levels of cholic acid, lithocholic acid and α-muricholic acid were significantly elevated in mice treated with APAP, while ursodeoxycholic acid and β-muricholic acid were significantly decreased. Lithocholic acid has previously been implicated in liver injury since the hydrophobic bile acids are inherently cytotoxic (Deo and Bandiera 2008). In a study of drug-induced perturbations in bile acid homeostasis, Yamazaki et al. (2013) evaluated 13 chemical compounds including APAP. Plasma, urine and liver tissues were profiled using untargeted MS methods, which detected both primary and secondary bile acids. Eight BAs were evaluated in urine, 15 in liver tissue extracts, and 17 in plasma samples. Similar changes were noted in the liver tissue extracts and plasma with an increase in the glycine conjugated BAs [glycocholic acid (GCA) and glycochenodeoxycholic acid (GDCA)] and cholic acid (CA) after APAP dosing. CA was also significantly increased in the urine samples and the increase was noted prior to visible histological changes and increases in ALT. Taurine-conjugated BAs were generally decreased after APAP treatment; the taurocholic acid (TCA) concentration in the high-dose APAP group on day 2 was elevated although not significantly. The decrease in TCA and the taurine conjugates could be related to decreases in taurine in the liver due to altered glutathione metabolism by APAP treatment. A systems biology study of APAP-induced hepatotoxicity in rats evaluated numerous serum biomarkers including bile acids by LC/QTof-MS and LC/TQ-MS (Sun et al. 2013). In general, the bile acids were decreased compared to control 6 h after dosing and then increased through 24–72 h, after which they tended to return to control values. CA, DCA and GCA were significantly elevated at 24 h after dosing in serum. Both TCA and TDCA had strong Pearson’s correlations to both log2ALT and hepatic necrosis scores. The authors hypothesized that a panel of bile acids including the plasma glycine and taurine conjugates and urinary CA may be able to detect drug-induced liver injury in rats. Select BAs, CA, GCA and taurocholic acid (TCA) were evaluated in the serum from rats dosed with various hepatotoxicants, including APAP and nonhepatotoxicants (Luo et al. 2014). The individual BAs were chosen based upon prior reports of CA, GCA and TCA as potential biomarkers of liver injury in clinical and nonclinical studies (Tribe et al. 2010; Trottier et al. 2011; Yamazaki et al. 2013). Rats were given a single dose of 1000 mg APAP/kg body weight; the dose was chosen to induce liver injury. Blood was collected at necropsy and processed to serum, which was analyzed using a LC/MS/MS method. APAP treatment resulted in significantly increased total serum BAs, CA, TCA and GCA. Yamazaki et al. (2013) similarly reported elevated CA and GCA as noted above. In contrast, the levels of TCA reported by Yamazaki et al. (2013) were generally decreased relative to control except on day 2 in the high-dose group. Since the current study collected serum on day 1, this may indicate that the increase in TCA occurs early and then cysteine is utilized for glutathione synthesis rather than for taurine biosynthesis, which results in decreased concentrations of the taurine conjugates. The serum from rats treated with APAP, which causes hepatocellular damage, showed the largest increase in CA, therefore leading the authors to hypothesize that a large increase in CA may be a potential marker for hepatocyte damage. Marked increases in TCA and GCA, conversely, may be markers of biliary injury. This study indicated the potential for total serum BA levels along with patterns of individual BAs is related to various types of liver injury. Table 3 reports seven BAs including CA, DCA, GCDC, GDCA, GCA, TDCA and TCA identified in the urine and blood of rats using MS methods (Kumar et al. 2012; Sun et al. 2013; Yamazaki et al. 2013) and further evaluated in clinical studies as described below.

Several clinical studies of the effects of APAP on total BA levels and individual BAs have also been reported. Table 3 reports the translation bile acid biomarkers of APAP-induced hepatotoxicity including the number of human subjects, biofluid and method validation method. James et al. (1975) noted that in 51 of 54 patients overdosed with APAP, the total serum BAs were elevated and the BAs appeared to be more sensitive to mild liver damage than the serum transaminase levels. A recent study employed a UPLC/MS method to evaluate the serum and plasma bile acids in patients hospitalized following APAP overdose (Woolbright et al. 2014). Total plasma bile acids were found to peak around day 1 after hospitalization and decreased over 6 days, but remained elevated compared to the control group. Six bile acids were evaluated in the plasma samples and followed the same trend noted for the total BAs, peaking on day 1 and subsequently declining although staying elevated compared with control. This suggested that these BAs could be predictive of the course of APAP-induced liver injury. GCA and TCA were measured as part of the panel of six BAs, and the results were consistent with the nonclinical data showing significant increases in the concentrations of these metabolites in the APAP patients compared to the control patients. The same bile acids measured in plasma were then measured in the serum of a more select cohort of the patients: patients with advanced drug-induced liver failure. Included in this group were survivors and nonsurvivors. Receiver operating characteristic (ROC) analysis was used to evaluate the ability of the individual bile acids to predict outcome. GDCA was significantly higher in the serum of nonsurvivors, and ROC analysis indicated that GDCA on the day of admission was somewhat predictive of death (area under the curve (AUC) of 0.70). ALT, on the other hand, was not significantly different between the two groups and did not predict severity of outcome after overdose. The authors also found that the glycine-amidated conjugates of the BAs and deoxycholic acid (DCA) increased more than the taurine conjugates, resulting in a more hydrophobic pool of BAs, which as noted above are more cytotoxic (Deo and Bandiera 2008). Bile acids have also been evaluated in serum samples from children and adolescents to discover APAP-induced hepatotoxicity biomarkers (Bhattacharyya et al. 2014a). Three subgroups were evaluated: a therapeutic APAP dose group, a control group and an APAP overdose group. A UPLC/TQ-MS method was employed for targeted analysis of nine serum BAs. Significant differences were found for six of the nine BAs measured in the APAP overdose group compared with control, while four of nine were significantly altered in the therapeutic dose group compared with control. Correlation analysis was performed to compare the BAs to the peak APAP protein adducts. Of the BAs measured, the highest correlation was found for GDCA, which was noted above to be the most predictive of outcome in the study by Woolbright et al. (2014). Both the therapeutic group and overdose group had significant elevations in GDCA compared to the control group. GDCA levels were also reported to be higher in those patients who had delayed treatment with NAC. Strong correlations were also noted for taurochenodeoxycholic acid (TDCA) and glycochenodeoxycholic acid (GCDC). TDCA was significantly elevated in both APAP groups, while GCDC was only significantly elevated in the overdose group. GCDC in the therapeutic group was elevated relative to control although not significantly. The results indicated that measurement of the total BA levels, GDCA, TDCA and GCDC, may serve as potential clinical markers of APAP hepatotoxicity.

### Pyroglutamic acid as biomarker of APAP-induced liver injury

Acute APAP hepatotoxicity has been reported as a common cause of metabolic acidosis, which sometimes occurs prior to liver injury (Flanagan and Mant 1986; Gray et al. 1987). The accumulation of pyroglutamic acid was recently reported as a rare and uncommon reason for metabolic acidosis in adults. In a previous study (Sun et al. 2013), pyroglutamic acid was increased at 24 h in both urine and serum from rats dosed with 1250 mg APAP/kg body weight. In 1990, Pitt et al. (1990) reported a pyroglutamic acidosis case which could be related to APAP use. Pitt el al. (1990) proposed that APAP consumption may have depleted GSH stores, which causes accumulation of pyroglutamic acid. Most recently, Duewall et al. (2010) also reported that a 39-year-old white woman had increased levels of pyroglutamic acid in urine, which could be related to chronic APAP use. In 2006, Fenves et al. (2006) reported four cases with pyroglutamic acidosis, and research of the literature identified 18 adult patients with the same syndrome. Twenty-one out of the 22 patients, all of whom had markedly elevated levels of pyroglutamic acid documented in urine and/or plasma, had taken APAP (only one was acute exposure to APAP). Table 3 reports the evaluation of pyroglutamic acid in a nonclinical study of APAP-induced hepatotoxicity (Sun et al. 2013) and its translational nature in two clinical studies (Duewall et al. 2010; Fenves et al. 2006). Similar cases with small number patients have also been reported (Dempsey et al. 2000; Foot et al. 2005; Humphreys et al. 2005; Pitt and Hauser 1998; Tailor et al. 2005; Yale and Mazza 2000). Although it is hard to ascertain the cause of pyroglutamic acidosis because most of the reported patients had multiple medical comorbidities and/or kidney dysfunction or kidney failure, all of the patients were using APAP. Resolution of pyroglutamic acidosis occurred after discontinuation of APAP. Lactic acidosis, another organic acid causing metabolic acidosis, has been reported at the early stage of or prior to APAP-induced liver injury in patients overdosed with APAP (Shah et al. 2011; Vichot and Rastegar 2014; Zein et al. 2010).

### Metabolomics APAP-induced liver injury biomarkers summary

It is well established that translation of metabolic biomarkers from animal studies to humans is challenging due to the species difference as well as the confounding factors in humans including diet, age, lifestyle, overall health, drug–drug interaction and others, all of which will influence the metabolome profile. Both long-chain acylcarnitines and BAs are sensitive to many of these other factors. Therefore, future research is needed to further validate acylcarnitines and BAs as clinical biomarkers of APAP toxicity. The nonclinical and clinical studies reported to date indicate that they could be predictive of the severity of APAP-induced injury, especially when measured in conjunction with other transcriptomics and proteomics biomarkers as well as the standard parameters.

## Final discussion and conclusions

Acetaminophen is the most widely studied hepatotoxic drug. As such, studies of APAP toxicity using clinical samples have the potential to generate data that will be of relevance to other studies of drug-induced liver injury. The recent introduction (Aronson 2005) of “omic” technologies allows for exploration of miRNA, protein and metabolites and represents a potentially promising approach for the evaluation of new biomarkers of drug toxicity and will refine translational systems medicine. This review was limited to biomarkers that have been examined to some extent in the clinical setting (Bailey et al. 2012; McGill and Jaeschke 2014) and to biomarkers that are amenable to measurement within biofluids that are easy to obtain in humans (e.g., blood and urine). While several biomarkers may represent strong potential candidates as early indicators of liver injury, the existing literature is limited by studies that have relatively small sample numbers and relatively sparse sampling strategies. Clinical toxicology studies are particularly challenging to conduct due to the significant variability in the clinical characteristics of the study patients. For example, accurate documentation of the dose of APAP ingested is extremely difficult, and APAP poisonings may be associated with ingestions of other medications. Furthermore, most studies are limited to daily sampling and thus were not designed to address rapid changes that may occur within the endogenous metabolome of the individual in response to high-dose ingestion of APAP. In addition, inclusion of information on treatment with NAC is an important component of these types of studies.

Despite the above challenges, recent publications suggest that the application of omics technology to the clinical setting of acute APAP toxicity can generate candidate biomarkers for further examination in future studies conducted with more optimized study designs. More frequent sampling, particularly in the early stages of the hospitalization, may generate more meaningful data that can compare the temporal profiles of candidate biomarkers to one another. It is important that biomarker samples are matched to samples that provide concurrent measurement of ALT and AST as well. In addition, APAP cases that did not develop liver injury, as well as patients who are receiving APAP at therapeutic doses are important controls that should be included in future clinical studies. Inclusion of patients with preexisting liver disease who are receiving low-dose APAP is also a comparison group for which little data exist. It is important to note that few studies have examined omics biomarkers in relationship to existing clinical criteria, such as coma or a constellation of clinical and laboratory findings (i.e., King’s College Criteria) currently in use by clinical practitioners as a prognostic indicator. Future studies should also consider the impact of nontoxic perturbations (exercise, diet, age, and other diseases and other organ toxicities) on biomarker performance.

The temporal response of potential translation biomarkers of liver injury such as acyl carnitines, miR-122 and proteins described in this review are dependent on toxicokinetics of APAP in each species. The toxicokinetics of APAP can be altered by genetics, dose, diet, age, alcohol and other factors, and these factors make translating biomarkers discovered in omics nonclinical studies to the clinic challenging. The response of translational biomarkers can be compared the current “gold standard” translational biomarker ALT for better understanding the limitations and potential application in clinical setting. APAP adducts are increased early and maintained at higher levels for longer times because kinetics of APAP adducts clearance from the subject is very slow. The kinetics of serum miR-122 appears to be closely following the response of the translational biomarker ALT, but more work is needed to determine this. The increase in palmitoyl carnitine levels in blood occurs before the maximum response in ALT and then palmitoyl carnitine usually decreases or is near normal during ALT maximum. Examination of translational biomarkers in clinical samples has the potential to elucidate mechanisms of drug toxicity that have not been previously identified. For example, the report of palmitoyl carnitine elevation as an acylcarnitine increased in APAP toxicity in mice led to clinical studies to measure this and other acylcarnitines in peripheral blood samples of APAP overdose subjects. The elevation of acylcarnitines provides some of the first data to demonstrate the clinical relevance of previous preclinical studies reporting a role for mitochondrial injury as a mechanism of APAP toxicity. Further examination of this and other metabolite profiles in the setting of chronic APAP exposures is indicated to better understand the potential impact of chronic drug therapy to disease expression and/or to alterations in endogenous metabolism. Finally, studies of miRNAs have the potential to provide new knowledge about drug-induced mechanisms of gene regulation that may have future relevance for translational systems medicine. If further testing continues to support the utility of these biomarkers to identify hepatotoxicity in other drugs besides acetaminophen in nonclinical species and in humans, the concept of biomarker qualification could be pursued for regulatory utility (US 2010).
